# TGF-β-induced α-SMA expression is mediated by C/EBPβ acetylation in human alveolar epithelial cells

**DOI:** 10.1186/s10020-021-00283-6

**Published:** 2021-03-04

**Authors:** Hui Ding, Jinjun Chen, Jingping Qin, Ruhua Chen, Zili Yi

**Affiliations:** 1 College of Bioscience & Biotechnology, Hunan Agricultural University, Changsha, 410128 Hunan China; 2 Department of Pulmonary and Critical Care Medicine, Yixing People Hospital, Affiliated to Jiangsu University, Yixing, 214200 Jiangsu China

**Keywords:** C/EBPβ, Fibrosis, Collagen, α-SMA, Pulmonary

## Abstract

**Background:**

Although the morbidity and mortality rates associated with idiopathic pulmonary fibrosis (IPF) are high, there is still lack of powerful and precise therapeutic options for IPF.

**Object:**

Through in vitro model, this study sought to determine whether binding of acetylated CCAAT/enhancer binding protein β (C/EBPβ) to alpha-smooth muscle actin (α-SMA) promoter could affect the activity of the latter as well as assess if it is essential for epithelial-to-mesenchymal transition (EMT) and extracellular matrix deposition in IPF.

**Methods:**

The expression of EMT and C/EBPβ in A549 cells treated with transforming growth factor-beta (TGF-β) as pulmonary fibrotic model was detected by western blotting and qPCR. Collagen-I expression using ELISA was performed. The luciferase activity was used to examine the activity of C/EBPβ. Knockdown of C/EBPβ was performed by siRNA. We also investigated the effect of deacetylation of C/EBPβ on EMT using sirtuin 1 (SIRT1). The binding ability of C/EBPβ with α-SMA promoter was affirmed via chromatin immunoprecipitation (ChIP) and electrophoresis mobility shift assay (EMSA). The relationship between α-SMA and acetylated C/EBPβ was determined with co-immunoprecipitation (Co-IP). SiRNA-mediated knockdown of C/EBPβ in A549 cells attenuated TGF-β1-induced myofibroblast differentiation and ECM deposition. The extent of association between acetylated C/EBPβ and α-SMA promoter was dynamically monitored.

**Results:**

It was confirmed that deacetylation of C/EBPβ in A549 cells successfully ameliorated TGF-β1-induced EMT, as shown by reduction in α-SMA expression and excessive collagen-I accumulation.

**Conclusion:**

The EMT and fibrotic effect of TGF-β1 is dependent on acetylated C/EBPβ-mediated regulation of α-SMA gene activity. Thus, C/EBPβ acetylation may play a central role in pulmonary fibrosis.

## Introduction

Idiopathic pulmonary fibrosis (IPF) is diagnosed as an irreversible progressive fibrotic disease with a median survival rate of up to 2.5−3.5 years after diagnosis [1]. The morbidity and mortality rates associated with IPF are high, while the abnormal pulmonary function of patients with IPF adversely affects their quality of life. Unfortunately, powerful and precise therapeutic options coupled with lung transplantation that are used to improve the prognosis for patients with IPF are lacking. Therefore, the investigation of the mechanisms of IPF pathogenesis is critical for the development of efficient IPF therapeutics.

The characteristic pathological feature of IPF is the usual interstitial pneumonia (UIP), marked by increased deposition of extracellular matrix (ECM) components such as fibronectin and collagen-I (Col-I) [2], which causes abnormality of lung tissue, resulting in pulmonary function insufficiency. Although the precise mechanism underlying the pathogenesis of lung fibrosis is unclear, accelerated proliferation of myofibroblasts has been shown to be possibly responsible for the excessive accumulation of ECM in the alveolar and interstitial compartments of the lung coupled with the development and prognosis of IPF [3, 4]. Previous studies have reported that epithelial-to-mesenchymal transition (EMT) due to injuries to alveolar epithelial cells (AECs) could contribute considerably to the initiation and maintenance of fibrosis [5, 6]. During EMT process, the epithelial cells lose cellular interaction and gain mesenchymal phenotype (myofibroblast-like) as well as the ability to generate ECM. Furthermore, reduced expression of E-cadherin, an epithelial cell marker and increased expression of α-smooth muscle actin (α-SMA), a biomarker for myofibroblast differentiation has been observed in fibrotic lung tissue [4]. Thus, regulation of α-SMA expression is critical for induction of myofibroblasts and genesis of fibrosis. Actually, inner and outer injuries to the epithelium could lead to the accumulation of cytokines and molecular sensors, which normally support the active EMT process, resulting in ECM deposition. Transforming growth factor-β (TGF-β) is known to be a major inducer of the initiation and maintenance of lung fibrosis [7]. Willis et al. reported that AECs that respond to TGF-β induced conversion to the mesenchymal phenotype, is associated with α-SMA-positive expression [8]. Hence, TGF-β1-induced EMT has been reported to play a dominant role in the pathology of pulmonary fibrosis.

Molecular signalling of TGF-β1-induced EMT is as complex as the activation of various pathways, including Smads [9], mitogen-activated protein kinase (MAPK) [10], and phosphatidylinositol 3-kinase (PI3K) [11]. In rodents, CCAAT/enhancer binding protein β (C/EBPβ), a leucine zipper transcription factor, has been shown to be localised in the AECs and bronchiolar epithelium using immunohistochemistry [12]. Other studies have shown that C/EBPβ is required for the maintenance of epithelial cells stability [13, 14]. Hu et al. reported that C/EBPβ activation was essential for cytokine secretion and differentiation of myofibroblasts in vivo [15]. Another study also demonstrated that mice lacking C/EBPβ antagonised the development of bleomycin-induced lung fibrosis [16]. Available evidence suggests that phosphorylation and acetylation result in changes in function of C/EBPβ including transcriptional activities of other genes [17]. Specifically, acetylation of C/EBPβ at lysine 39 modulated its capability to activate transcription [18]. Likewise, phosphorylation of C/EBPβ on numerous residues in many cases could lead to an increase in its transcriptional activity [19]. Nonetheless, the post-translational modification of C/EBPβ responsible for activation of α-SMA expression is still unclear.

Schwartz et al. confirmed that acetylation of C/EBPβ is necessary for cytokine activation and downstream transcription [18]. Based on this assertion, we hypothesised that acetylation of C/EBPβ could induce the binding of the α-SMA promoter, which may subsequently activate gene expression that is critical for ECM deposition and lung fibrosis. To affirm this hypothesis, we treated the human alveolar epithelial cell line A549 with TGF-β1 to establish an in vitro model of lung fibrosis. The level of C/EBPβ acetylation associated with the α-SMA promoter to indicate the EMT progression was dynamically monitored. Furthermore, siRNA-mediated knockdown of C/EBPβ in A549 cells attenuated TGF-β1-activated myofibroblast differentiation and ECM deposition. We therefore postulated that deacetylation of C/EBPβ in A549 cells may successfully ameliorate TGF-β1-induced EMT via decrease in α-SMA expression, followed by reduction in excess collagen-I accumulation, a characteristic of lung fibrosis.

## Methods

### Cell culture and siRNA stimulation

Human alveolar epithelial A549 cells were purchased from ScienCell^®^ and propagated as described previously [20]. The A549 cells were plated in six-well plates and grown overnight under serum deprivation till 70% confluence was reached. For knockdown experiments, the A549 cells were pre-treated with C/EBPβ small interfering RNAs (C/EBPβ siRNA) (10 nM) according to the manufacturer’s protocol (Santa Cruz, USA) for 24 h. Exogenous sirtuin 1 (SIRT1, R&D Systems, USA). For C/EBPβ deacetylation, a class III histone deacetylase was added to the cells at the final concentration (of 2 µM) 24 h prior to the addition of 10 ng/mL TGF-β (R&D Systems, Cat. 240B-010). All the experiments were performed in A549 cells between passages 4 and 8. Whole-cell lysates were prepared for protein and mRNA collection.

### Western blot analysis

The proteins isolated from A549 cell lysates were quantified using the bicinchoninic acid (BCA) method. Equal amounts (60 µg) of protein were electrophoresed on 12% sodium dodecyl sulphate-polyacrylamide gel and transferred to polyvinylidene fluoride (PVDF) membrane. The membranes were incubated overnight with specific antibodies against glyceraldehyde-3-phosphate dehydrogenase (GAPDH) (R&D Systems, Cat. 2275-PC-100), acetylated-C/EBPβ (Ac-C/EBPβ; Santa Cruz, USA,Cat. Sc-365546AC), C/EBPβ (Abcam, USA, Cat. ab32358), and α-SMA (Abcam, Cat. ab7817) at 4 °C and then incubated with appropriate secondary antibodies at room temperature for 4 h. The chemiluminescence signals developed via the enhanced chemiluminescence (ECL) kit (Santa Cruz) was quantified using the Quantity One software. All the independent experiments were repeated thrice.

### Real time-polymerase chain reaction (RT-PCR)

Total RNA was extracted from A549 cells using the RNA extraction kit (Lucigen, USA) according to the manufacturer’s instruction and was quantified using a spectrophotometer. The integrity of RNA (300 ng) was determined for each assay. The sequences of the primers (Applied Biosystems Inc., Foster City, CA, USA) are as follows: C/EBPβ forward 5′-GCCTCTCCACGTCCTC-CTCGT-3′ and reverse 5′-CACCTTCACCGTTCCAGTTT-3′; α-SMA forward 5′-GTGACTACTGCCGAGCGTG-3′ and reverse 5′-ATAGGTGGTTTCGTGGATGC-3′; forward 5′-CACCTTCACCGTTCCAGTTT-3′ and reverse 5′-CTCTTCCAGCCTTCCTTCCT-3′. RT- PCR was performed for 30 cycles of 51 °C for 30 min, 95 °C for 30 s, 94 °C for 45 s, 60 °C for 45 s, and 72 °C for 10 min in a GeneAmp 7500 sequence detection system (Applied Biosystems Inc.). The Taqman One Step RT-PCR master mix was used. The PCR products were electrophoresed on a 2% agarose gel and data were quantified with GAPDH as the internal control using the UVP bioimaging system GDS-8000. The mean Ct value of triplicate experiments was used for data calculation.

### Chromatin immunoprecipitation (ChIP) assays

The A549 cells exposed to TGF-β for 24 h were prepared for ChIP assays using a high sensitivity ChIP assay kit (Abcam) according to the manufacturer’s instructions. A PCR was performed using the following primers: 5′-GCT GTC GTV TTA TCT CCA CCA-3′ and 5′-GCA GGA GTC TAG CAG AAG TTC-3′. All the data were collected from three independent studies.

### Co-immunoprecipitation (Co-IP)

Protein samples were immunoprecipitated with either polyclonal antibody against C/EBPβ (Abcam) or control IgG (Santa Cruz) at 4 °C overnight. Next, the samples were constantly agitated with A/G-agarose beads (Santa Cruz) at 4 °C for 4 h. After five washes with the buffer, the beads were used for protein extraction. Proteins from deposits with lysate were used for western blotting as mentioned above. The antibodies used for western blotting were anti-acetylated-lysine antibody (Santa Cruz, sc-32268) and anti-α-SMA (Abcam, Cat. ab7817).

### Enzyme-linked immunosorbent assay (ELISA)

The levels of collagen-I were measured in cell-free supernatants from A549 cells after stimulation with factors using ELISA kits (R&D Systems) in accordance with the manufacturer’s protocol. The data were obtained from three separate experiments.

### Luciferase reporter assay

The luciferase activity assay was performed as described previously [21]. Briefly, luciferase reporter gene constructs under the control of the 5′-flanking promoter sequence of the human α-SMA gene with the conserved putative C/EBPβ-binding motif TTGGGCAA was constructed by inserting DNA fragment and generated via PCR into the pGL4.10 vector (Promega, Madison, WI). The A549 cells in 12-well plates were treated with C/EBPβ-Luc (0.8 µg) or 0.5 µg α-SMA-Luc from Stratagene (USA) for 24 h and were subsequently used for luciferase reporter assays. The cells were treated with exogenous sirtuin 1 (SIRT1, 2 µM) for 24 h as described in earlier work with some modifications [22]. Next, the cells were activated with or without TGF-β (10 ng/mL) for 16 h. The luciferase activity of the stimulated cells was measured using the luciferase assay system (Promega, USA).

### Statistical analysis

All data were presented as mean ± standard error of mean (SEM). For the significance between two group samples, a two-tailed parametric or non-parametric t-test was used. Two-way analysis of variance (ANOVA) followed by Bonferroni’s test was performed for multiple comparison between more than two groups. The control samples from separate experiments were normalised to a value of one. A P-value < 0.05 was considered statistically significant.

## Results

### TGF-β induces EMT of A549 cells

**Fig. 1:**
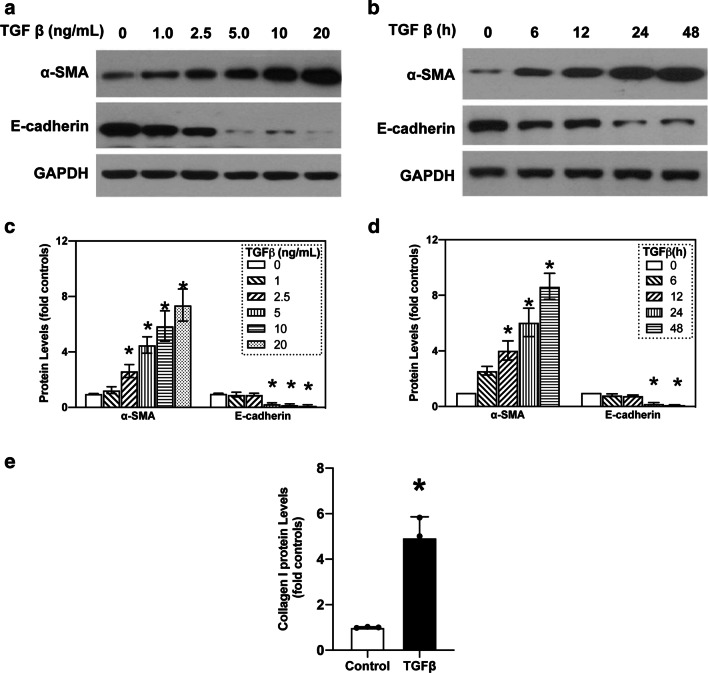
TGF-β induced EMT and deposition of collagen-I in A549 cells. **a** A549 cells were exposed to TGF-β (0−20 ng/mL) for 24 h. Protein levels of α-SMA and E-cadherin were determined using western blotting, and quantification of western blots were calculated according to grey analysis. In agreement with the elevated expression of α-SMA, E-cadherin expression decreased in a dose-dependent manner, compared to non-stimulated cells. **b** Expression of α-SMA and E-cadherin in A549 cells treated with TGF-β (10 ng/mL) for 0, 6, 12, 24, and 48 h was assessed using western blotting. **c**, **d** Quantification of western blots were calculated according to analysis. **e** ELISA analysis of collagen-I levels in the supernatant of A549 treated with 10 ng/mL TGF-β for 24 h or controls. All data were from three separate experiments. Asterisk (*) denotes significant difference (P < 0.05)

Based on previous work [23], the A549 cells were first treated with various amounts of TGF-β at different time periods to determine the safe and optimal concentration of TGF-β as well as the incubation time required for the investigation of EMT in vitro. When A549 cells were treated with 1.0, 2.5, 5.0, 10 and 20 ng/mL TGF-β for 24 h, we observed a dose-dependent increase in the expression of the mesenchymal marker α-SMA, while a decrease in the expression of epithelial biomarker E-cadherin was identified via western blotting (Fig. 1a, Additional file 1: Fig. S1A), which collectively suggests mesenchymal differentiation. Although α-SMA level increased in A549 cells after treatment with 20 ng/mL TGF-β, cell viability was poor. In addition, A549 cells were treated with 10 ng/mL TGF-β at different time periods (0, 6, 12, 24, and 48 h) to determine the appropriate time frame of treatment. We observed an increase in the expression of α-SMA in a time-dependent manner, which peaked at 48 h by 8.72 ± 0.45-fold compared with that of the control, but cell death was evident. E-cadherin expression decreased significantly in a time-dependent manner after 24 h of exposure to TGF-β (Fig. 1b, Additional file 1: Fig. S1B). Therefore, the optimal TGF-β concentration and incubation time were 10 ng/mL and 24 h, respectively. The level of collagen-I (determined using ELISA) increased upon A549 cells exposure to TGF-β (Fig. 1d), suggesting that an in vitro model of EMT-induced fibrosis was successfully established by treating A549 cells with optimal concentration of TGF-β for specific exposure duration.

### Up-regulation of C/EBPβ is involved in TGF-β-induced EMT

**Fig. 2:**
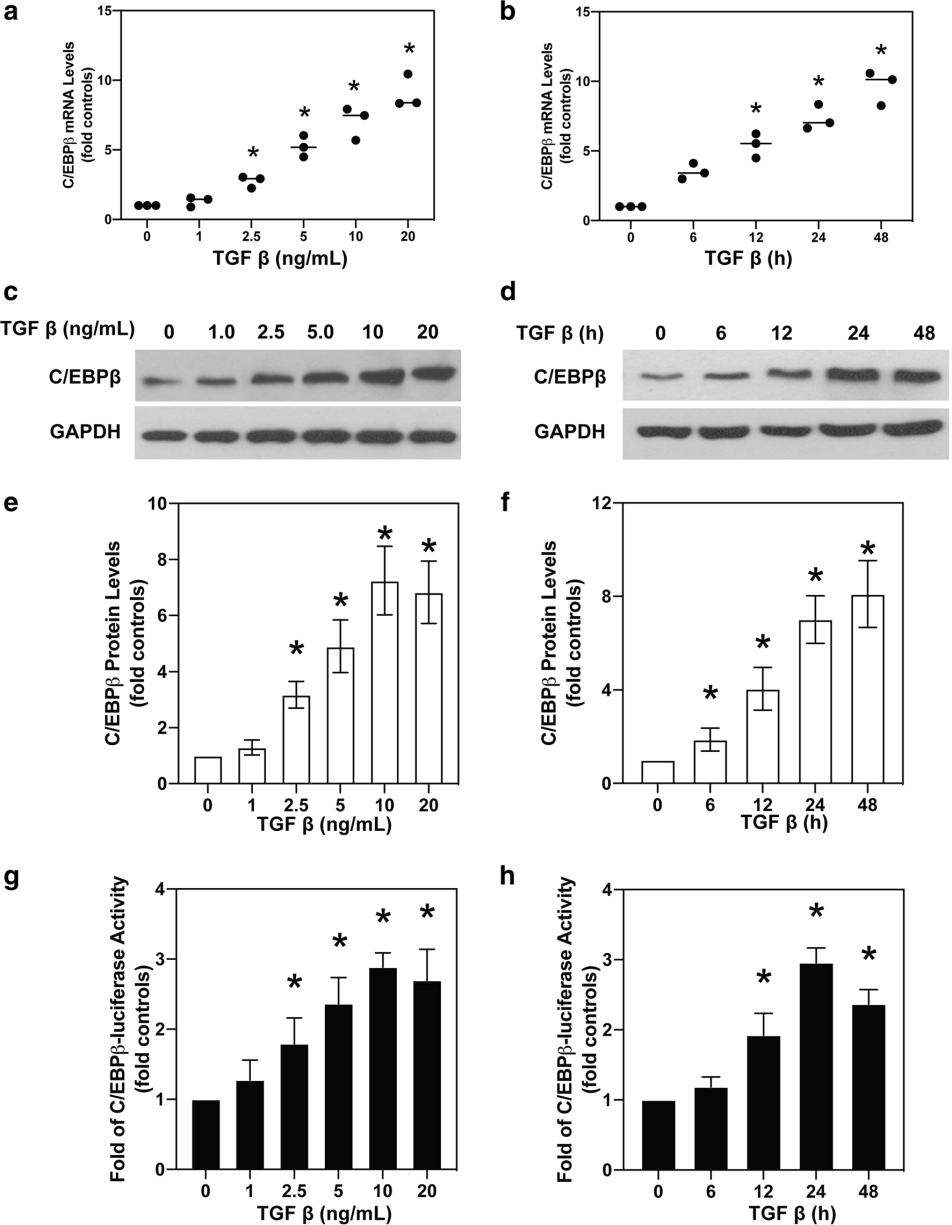
Up-regulation of C/EBPβ is involved in TGF-β-induced EMT. **a** Real-time PCR analysis of C/EBPβ mRNA levels in A549 cells treated with 0−20 ng/mL TGF-β for 24 h. **b** C/EBPβ mRNA levels increased in a time-dependent manner of TGF-β (10 ng/mL) treatment (0−48 h) in A549 cells. **c** TGF-β increased C/EBPβ protein levels in A549 cells in a concentration- (0, 1, 2.5, 5, 10, and 20 ng/mL) dependent manner. **d** C/EBPβ expression increased gradually by 7.05 ± 0.81 folds by 24 h when TGF-β treatment was prolonged. **e**, **f** Quantification of western blots were calculated according to grey analysis. **g** Luciferase activity assay was performed to assess C/EBPβ promoter activity. Generation of reporter gene constructs controlled by the human α-SMA gene promoter. Reporter constructs were generated by inserting fragments of the α-SMA gene promoter into a luciferase reporter vector pGL4.10. The location of the C/EBPβ-binding motif is as indicated. Relative to the control, the activity of C/EBPβ-luciferase increased with TGF-β concentration and peaked at 10 ng/mL by 2.74 ± 0.42-fold. **h** TGF-β up-regulated C/EBPβ-luciferase activity. Traces represent three experiments with similar results. Means ± SEM of data are shown. Asterisk (*) denotes significant difference (P < 0.05)

Previous study has shown that phosphorylation of C/EBPβ is involved in pulmonary fibrosis in mice [24]. To investigate the precise roles of C/EBPβ in fibrosis, the relationship between C/EBPβ activation and TGF-β-induced EMT was assessed. In this study, we observed that TGF-β up-regulated C/EBPβ mRNA expression level in dose-and time-dependent manner (Fig. 2a, b). Furthermore, western blot analysis showed that the expression levels of C/EBPβ in A549 cells increased with dose-and time of TGF-β treatment at 10 ng/mL within 48 h (Fig. 2c, d, and Additional file 2: Fig. S2C, D). The TGF-β-mediated increase in C/EBPβ expression was obvious when EMT was induced in the A549 cells. Next, we employed luciferase reporter assay to better understand C/EBPβ activation after TGF-β-induced EMT. Exposure of A549 cells to TGF-β generated time-and dose-dependent increase in C/EBPβ-luciferase activity and exhibited a 2.83 ± 0.42-fold increase in expression compared with that of the control (Fig. 2e, f). Collectively, these observations suggested that TGF-β increased the expression and activation of C/EBPβ during the EMT (Additional file 3: Fig. S3).

### Loss of C/EBPβ shifts TGF-β-induced collagen deposition following EMT

**Fig. 3:**
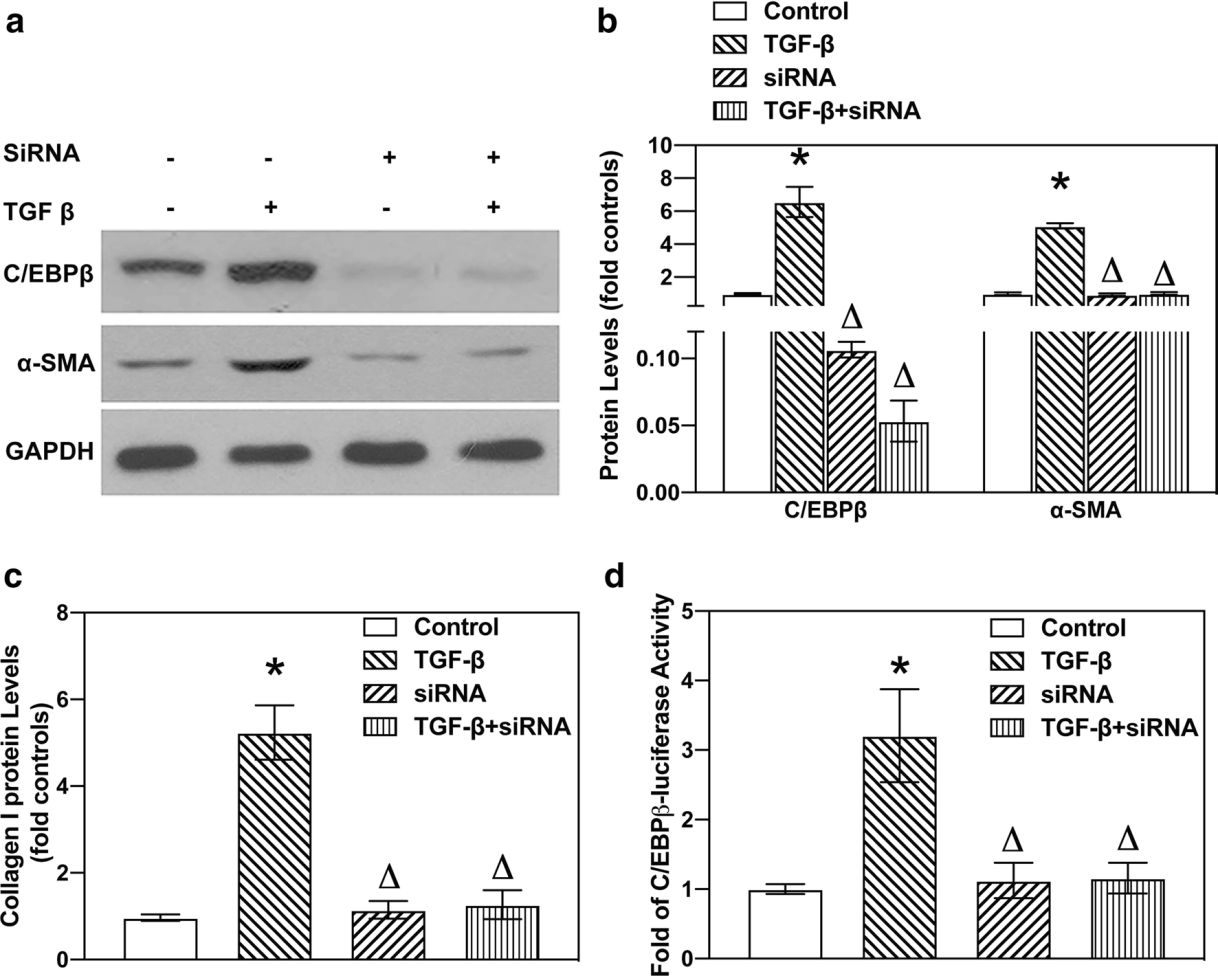
TGF-β up-regulated the expression of α-SMA via C/EBPβ activation in A549 cells. **a** TGF-β increased α-SMA protein levels via C/EBPβ activation. A549 cells were treated with TGF-β (10 ng/mL) for 24 h with or without the presence of C/EBPβ siRNA (10 nM) for 8 h. Levels of C/EBPβ and α-SMA in cell lysate were analysed using western blotting. **b** Quantification of western blots were calculated according to grey analysis. **c** The C/EBPβ siRNA blocked TGF-β-induced increase in collagen-I synthesis. The effect of C/EBPβ siRNA on collagen-I deposition in TGF-β-treated A549 cells was analysed using ELISA. **d** TGF-β-induced C/EBPβ-luciferase activity is regulated by the C/EBPβ siRNA. A549 cells transfected with C/EBPβ siRNA successfully attenuated TGF-β-activated C/EBPβ-luciferase activity. Asterisk (*) denotes significant difference (P < 0.05)

Previous studies have established the involvement of C/EBPβ activation in the pulmonary fibrotic process [15, 16]. Other studies have also reported that mice with C/EBPβ deficiency antagonise BLM-induced pulmonary fibrosis in vivo [15]. In this regard, we investigated whether C/EBPβ is required for TGF-β-activated EMT and collagen-I deposition. Next, C/EBPβ siRNA (10 nM) was used to establish the reducing gene model in A549 cells. As shown in Fig. 3a, the C/EBPβ siRNA successfully decreased gene expression. Also, the A549 cells transfected with the C/EBPβ siRNA attenuated TGF-β-induced α-SMA and collagen-I expression (Fig. 3a, b, and Additional file 4: Fig. S4). Furthermore, TGF-β could not increase C/EBPβ-luciferase activity in A549 cells treated with the C/EBPβ siRNA (Fig. 3c). Taken together, our results suggest that C/EBPβ may be a crucial factor for the regulation of TGF-β-induced EMT and collagen-I deposition in pulmonary fibrosis.

### TGF-β induced C/EBPβ binding to α-SMA promoter in A549 cells

**Fig. 4:**
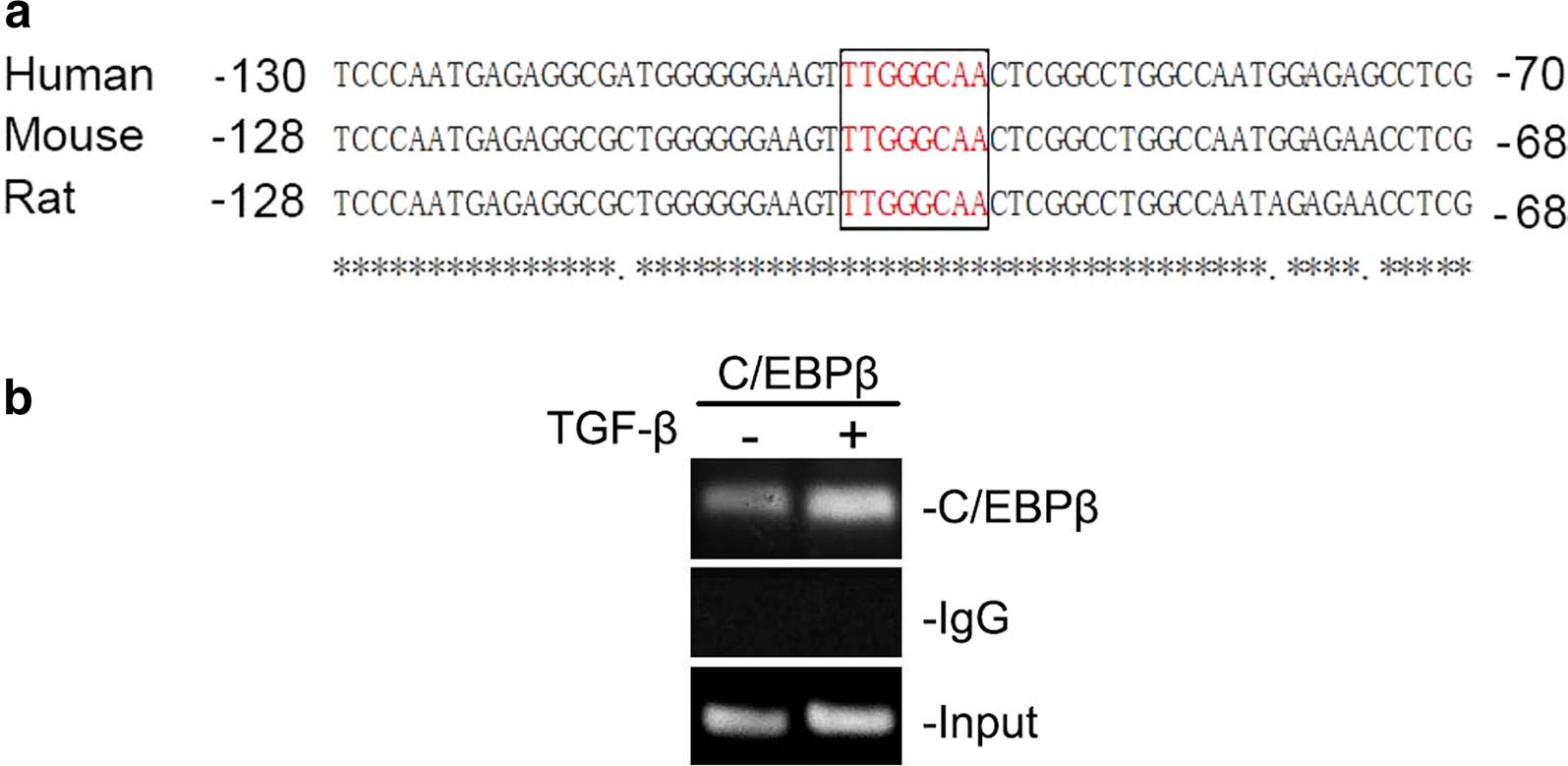
TGF-β induced C/EBPβ binding to α-SMA promoter in A549 cells. **a** The 5′ promoter sequence of the human, mouse and rat α-SMA gene contains a putative C/EBPβ-binding motif (TTGGGCAA) within 200 bp from the transcription start site. *Indicates conserved sequences. **b** A549 cells were exposed to TGF-β (10 ng/mL) for 3 h, followed by ChIP assay. ChIP primer pairs, yielding 237-bp PCR products, were designed to amplify DNA corresponding to the C/EBPβ-binding site. A rabbit IgG clone was used as a negative control. Typical traces are representative of two experiments with similar results

As mentioned above, our data confirmed that C/EBPβ played a pivotal role in EMT and pulmonary fibrosis in vitro. As an important transcription factor, C/EBPβ triggered the expression of downstream genes by binding to its cognate sites in the gene promoters. However, the C/EBPβ binding site on the α-SMA promoter region in A549 cells is not known. Therefore, to elucidate the molecular mechanism through which TGF-β regulates α-SMA expression, we explored the putative C/EBPβ-binding sites in the 5′ promoter region of human α-SMA gene. Also, the 5′ promoter region of the human, mouse and rat α-SMA gene was examined via Multiple Sequence Alignment. We found a conserved putative C/EBPβ-binding motif TTGGGCAA in the 5′ promoter region within 200 bp from the identified transcription start site (Fig. 4a). We therefore hypothesised that the putative C/EBPβ-binding motif was a C/EBPβ-responsive cis-element that mediates the upregulation of the α-SMA gene, and that the activation of this cis-element is critical for the development of EMT. To test our hypothesis, we evaluated C/EBPβ binding to the putative binding motif present in the α-SMA promoter in A549 cells through the ChIP assay. We observed that TGF-β-treated A549 cells showed increased C/EBPβ binding to the α-SMA promoter region (Fig. 4b, Additional file 4: Fig. S4). Altogether, these results suggested that activated C/EBPβ could accelerate TGF-β-induced EMT by binding to the α-SMA promoter region in the A549 cells.

### Role of acetylation of C/EBPβ in binding to the α-SMA promoter in A549 cells

**Fig. 5:**
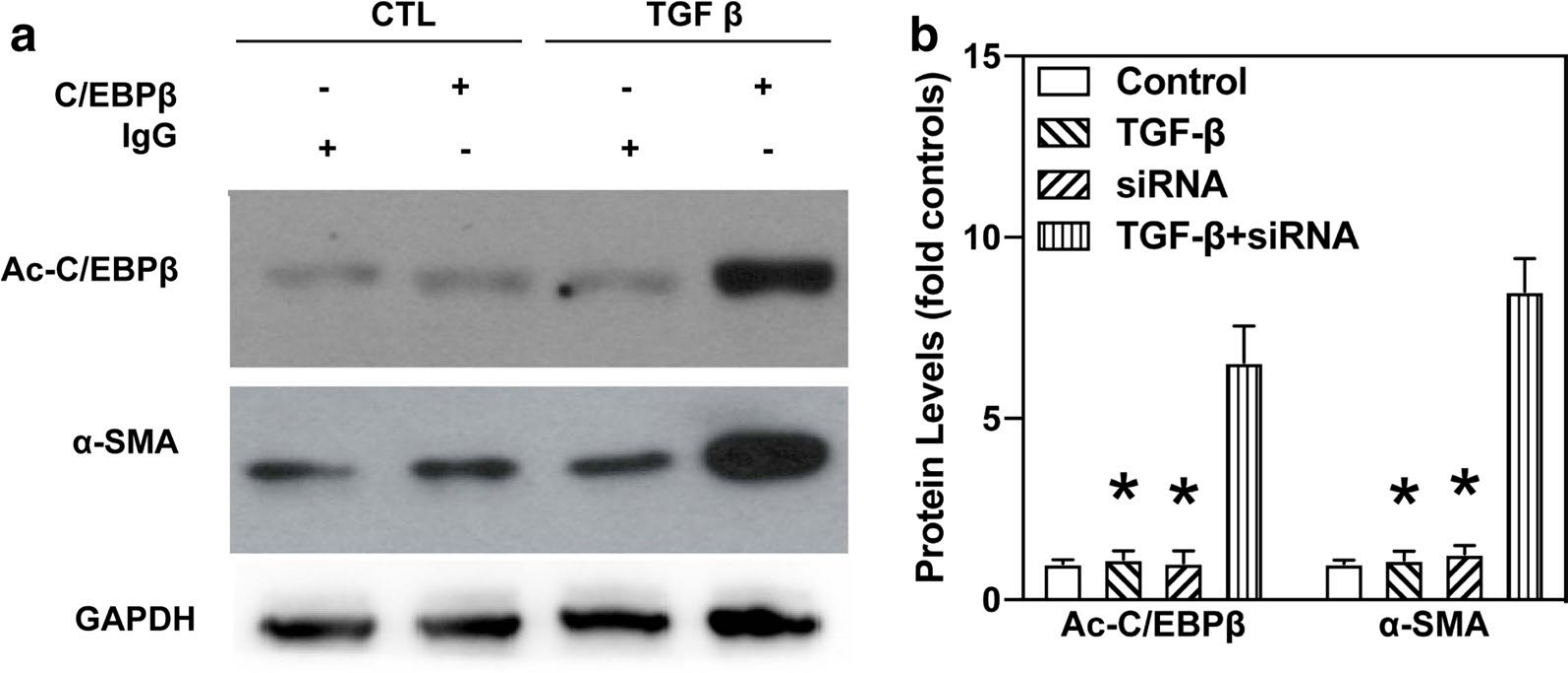
Involvement of C/EBPβ acetylation in TGF-β-induced EMT in A549 cells. **a** Cell lysates were prepared from A549 cells treated with 10 ng/mL TGF-β for 24 h. After treatment with the C/EBPβ antibody (described in “Methods”), C/EBPβ acetylation was shown to be involved in TGF-β-induced α-SMA expression. **b** Quantification of western blots were calculated according to grey analysis. However, elevated expression of acetylated C/EBPβ and α-SMA proteins was not observed in A549 cells treated with IgG during immunoprecipitation. Data show mean ± SEM. Asterisk (*) denotes significant difference (P < 0.05) compared to the control

Other reports have shown that phosphorylation of C/EBPβ plays a critical role in alveolar EMT and acts an essential step in pulmonary fibrosis [25, 26]. As a transcription factor, C/EBPβ activates its downstream signals via post-translational modification, such as phosphorylation, acetylation and methylation. However, reports on the role of C/EBPβ acetylation in pulmonary fibrosis are lacking. As shown above, activated C/EBPβ binds to the α-SMA promoter region in TGF-β-treated A549 cells. Nonetheless, the mechanism underlying C/EBPβ binding to α-SMA expression is not clearly understood. Hence, we investigated the effect of TGF-β on C/EBPβ modification and observed that acetylation of C/EBPβ could increase significantly by 6.42 ± 0.72-fold in the A549 cells treated with TGF-β (Fig. 5, Additional file 5: Fig. S5). An enhanced α-SMA expression was observed in samples treated with the C/EBPβ antibody but not in TGF-β-treated cells with immunoglobulin G (IgG). Collectively, our results showed that α-SMA expression may be triggered by C/EBPβ activation through acetylation.

### Acetylation and deacetylation of C/EBPβ in TGF-β-induced EMT and collagen deposition

**Fig. 6:**
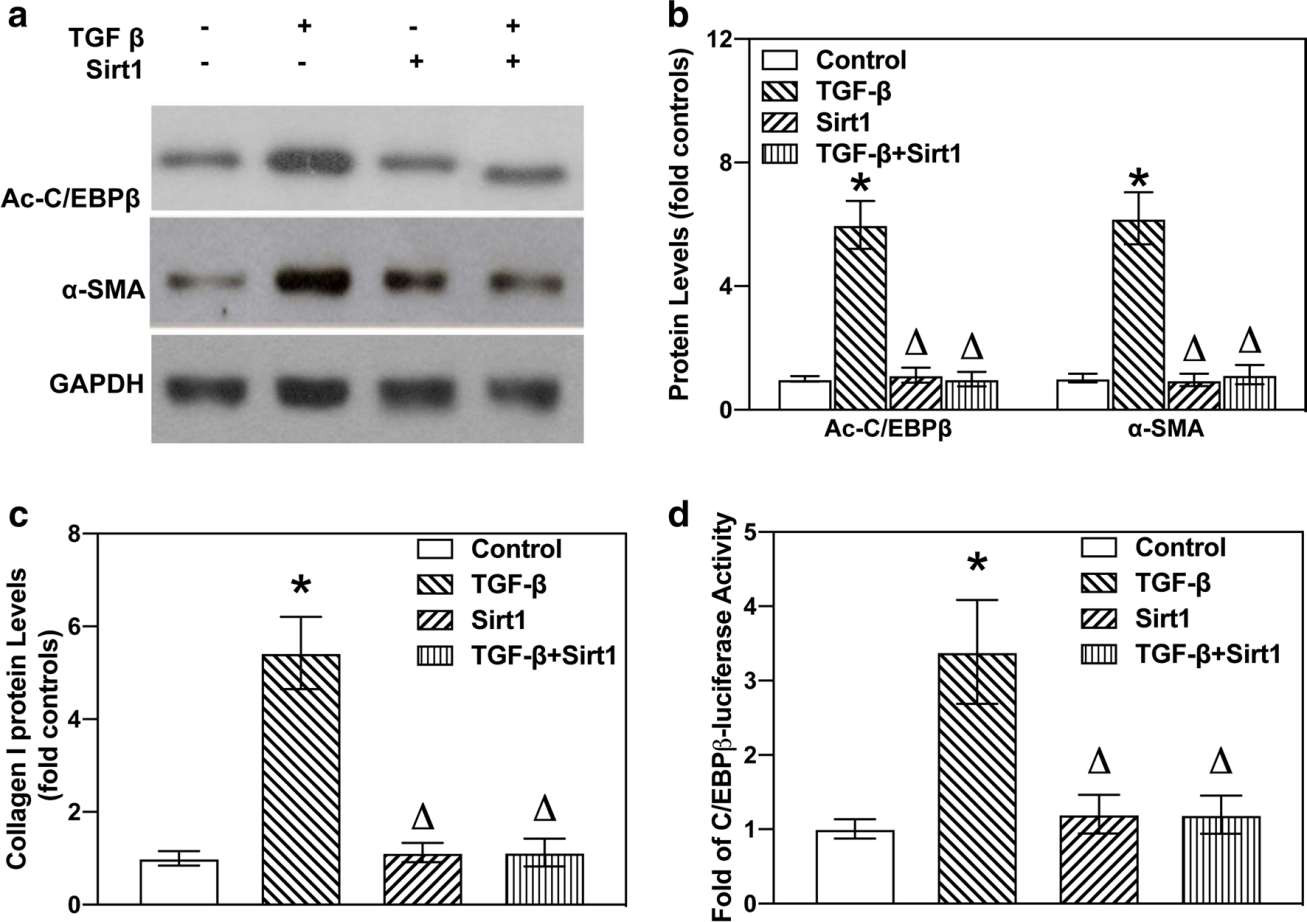
Roles of C/EBPβ deacetylation in TGF-β-induced EMT and collagen deposition. **a** A549 cells were treated with TGF-β (10 ng/mL) for 24 h. Western blotting showing TGF-β treatment led to acetylation of C/EBPβ and increased the levels of α-SMA proteins. **b** Deacetylation of C/EBPβ via SIRT1 administered in A549 cells rectified TGF-β-induced elevation in collagen-I deposition. **c** Quantification of western blots were calculated according to grey analysis. **d** SIRT1 suppressed the increased C/EBPβ-luciferase activity in TGF-β-treated A549 cells. Data show mean ± SEM. Asterisk (*) denotes significant difference (P < 0.05) compared to the control

Herein, we used TGF-β-treated A549 cells to mimic the pulmonary fibrotic model in vitro. The binding of C/EBPβ to α-SMA coupled with the increased expression and acetylation of C/EBPβ indicated that acetylated C/EBPβ may be involved in EMT and pulmonary fibrosis. As demonstrated in Fig. 6a, TGF-β treatment led to acetylation of C/EBPβ and accelerated EMT-induced collagen-I deposition. Acetylation of C/EBPβ could be important for its activation. To clarify whether acetylation of C/EBPβ is necessary for pulmonary fibrosis, we subsequently investigated the effect of deacetylation of C/EBPβ on EMT using SIRT1. As reported, SIRT1, a class III histone deacetylase (HDAC), specifically deacetylates histone or non-histone proteins, while the C/EBPβ is one of the deacetylation targets of SIRT1 [27]. We observed that SIRT1 could reverse TGF-β-induced C/EBPβ acetylation (Fig. 6a). Importantly, C/EBPβ deacetylation significantly reversed the elevated expression of α-SMA and collagen-I in TGF-β-treated A549 cells (Fig. 6a, b). As indicated earlier in this work, SIRT1 stimulation also suppressed the increased C/EBPβ-luciferase activity in TGF-β-treated A549 cells (Fig. 6c). These observations suggested that acetylation and deacetylation are useful steps in regulating C/EBPβ functions. All these results confirm that C/EBPβ acetylation may be a key player in alveolar EMT and that pulmonary fibrosis is blocked by its deacetylation.

## Discussion

Induction of C/EBPβ phosphorylation corresponds to lung fibrosis in mice [15]. In this study, we demonstrated that TGF-β leads to acetylation of C/EBPβ, which in turn enhances its activation and up-regulates collagen-I deposition. Furthermore, acetylation of C/EBPβ accelerates EMT in A549 cells by activating α-SMA after binding to the α-SMA promoter. In addition, C/EBPβ deficiency attenuates α-SMA and collagen-I production. Deacetylation of C/EBPβ is a major hurdle for TGF-β-induced EMT and collagen-I synthesis. Our observations revealed that acetylation/deacetylation of C/EBPβ play a central role in EMT and possibly in pulmonary fibrosis via α-SMA activation.

A growing body of evidence shows that myofibroblasts are the main players involved in extracellular matrix (collagen I) production during IPF [28]. Multiple factors stimulate the EMT of lung epithelial cells, which may contribute significantly to myofibroblast formation in pulmonary fibrosis. During EMT, lung epithelial cells lose cellular polarity and gain migratory properties; this is accompanied by the deficiency of the epithelial marker E-cadherin and acquisition of the mesenchymal marker α-SMA [29, 30]. Hence, EMT is studied by determining changes in the expression of these two markers and other biomarkers such as vimentin and N-cadherin. However, this work evaluated the successful induction of EMT using α-SMA and E-cadherin since there is co-expression of both markers during the conversion of epithelial cells to mesenchymal-like myofibroblasts [31]. Herein, we used TGF-β-treated A549 cells to mimic pulmonary fibrosis in vitro. Along with the reduction in E-cadherin expression, TGF-β improved α-SMA expression in a dose- and time-dependent manner. With the reduction in E-cadherin expression and increase in α-SMA expression, A549 cells gradually transformed into myofibroblasts, which secrete excess collagen-I, resulting in pulmonary fibrosis [32]. Therefore, blockage of α-SMA expression is a promising way of inhibiting EMT in pulmonary fibrosis. The molecular mechanism through which α-SMA regulates fibrosis has been the focus of many investigations. In this study, the TGF-β-treated A549 cells provided a credible in vitro model for studying these mechanisms.

As a critical transcription factor, C/EBPβ expression in the alveolar and bronchiolar epithelium of rodents significantly affects cellular proliferation and differentiation [33]. In vivo data confirmed that mice with C/EBPβ deficiency showed significant attenuation of bleomycin-induced myofibroblast accumulation and pulmonary fibrosis [16]. Available evidence suggests that hypoxia-induced expression of connective tissue growth factor (CTGF), resulted in pulmonary fibrosis, and was dependent on C/EBPβ activation [21]. Besides, a previous study confirmed that C/EBPβ was a key mediator of TGF-β-dependent fibroblast remodelling in asthma [34]. Likewise, C/EBPβ may play a pivotal role in the development of pulmonary fibrosis. Yuka et al. observed that TGF-β regulated the target genes of C/EBPβ, which indicated that C/EBPβ was a potential downstream effector of TGF-β signalling [6]. Also, augmented expression of the TGF-β receptor 2 in embryonic stem cells with over-expression of C/EBPβ indicated the reciprocal relationship between TGF-β and C/EBPβ signalling [35]. Another previous study has shown that TGF-β regulated cellular growth, invasion and metastasis depended on C/EBPβ activity in the mammary epithelial cells [36]. However, the involvement of C/EBPβ in EMT of lung is unclear. Thus, we reasoned that C/EBPβ may act as a key player in TGF-β-induced EMT in human alveolar epithelial cells. In this work, we observed that TGF-β increased C/EBPβ mRNA and protein levels. Besides, the enhanced activity of C/EBPβ-luciferase in TGF-β-treated A549 cells strengthened the evidence supporting the existence of a TGF-β-C/EBPβ signalling pathway. Also, we observed that the C/EBPβ siRNA suppressed TGF-β-induced EMT, as observed by reduction in α-SMA expression and collagen-I synthesis. Our study reveals that C/EBPβ is required for profibrotic processes in TGF-β-treated A549 cells. Altogether, activation of C/EBPβ-dependent α-SMA may be involved in TGF-β-induced pulmonary fibrosis.

Evidence suggests that C/EBPβ regulates signalling by binding to its cognate sites in target genes. Chen et al. reported that hypoxia-induced CTGF-luciferase activity depended on the binding of C/EBPβ to the ADAM 17 promoter site in human lung fibroblasts [21]. A related study showed that the binding of C/EBPβ to the α-SMA promoter was involved in interleukin 1 (IL-1)-regulated inflammation in rat lung myofibroblasts [37]. However, C/EBPβ binding to the α-SMA promoter in pulmonary fibrosis had not been previously investigated. In this work, we demonstrated binding of C/EBPβ to the α-SMA promoter in A549 cells. On the other hand, TGF-β promoted the expression of α-SMA in human alveolar epithelium, which was dependent on C/EBPβ. The above studies clearly indicated that TGF-β-induced EMT was dependent on the binding of C/EBPβ to α-SMA promoter sites.

The C/EBPβ binding to this region of α-SMA promoter indicates that C/EBPβ may block TGF-β-activated α-SMA expression, albeit the specific pathway remaining unclear. As a transcription factor, C/EBPβ activation is regulated by multiple mechanisms of post-translational modification, including phosphorylation and acetylation. Indeed, human C/EBPβ has several known phosphorylation sites, including Thr235, Thr266 and Thr273, which are important for intracellular localisation and transcriptional activity. An in vivo study demonstrated that C/EBPβ phosphorylation on Thr217 contributed to bleomycin-induced lung fibrosis in mice [24]. Chen et al. observed that C/EBPβ and C/EBPβ-luciferase activity, which depended on its phosphorylation, was involved in hypoxia-activated lung fibrosis [21]. A recent study revealed that regulation of artificial C/EBPβ phosphorylation may ease the membrane damage in acute lung injury (ALI) and improve membrane repair [14]. Chromatin remodelling is an essential mechanism that regulates gene transcription while acetylation/deacetylation plays pivotal roles in the functioning of several transcription factors [38, 39]. The mechanism through which acetylation/deacetylation regulates C/EBPβ activity has been described previously. Huang et al. observed that C/EBPβ acetylation by p300, a nuclear co-activator with intrinsic acetyltransferase activity, plays a key role in inflammatory responses in human lung epithelial cells [40]. However, the roles of C/EBPβ acetylation in lung fibrosis are unclear. Hu et al. identified the binding of C/EBPβ to the α-SMA promoter in rat lung fibroblasts [37]. We proposed that acetylation of C/EBPβ may determine the outcome of collagen-I expression and α-SMA activation in TGF-β-treated A549 cells. Our results showed that acetylation of C/EBPβ were involved in TGF-β-induced EMT and lung fibrosis in vitro. Results of the luciferase reporter assay showed that C/EBPβ activity was moderated by the extent of acetylation. Furthermore, using Co-IP, the enhanced expression of α-SMA in TGF-β-treated A549 cells was shown to depend on C/EBPβ activation. To understand the importance of C/EBPβ acetylation in α-SMA gene expression and lung fibrosis, we used SIRT1, a class III histone deacetylase, to construct a cellular model of C/EBPβ deacetylation in vitro. We observed that enhanced C/EBPβ acetylation and C/EBPβ-luciferase activity caused by TGF-β treatment was reduced in A549 cells after SIRT1 administration. Probably, Sirt1 could deacetylate C/EBPβ intracellularly through its ability to dynamically shuttle between cytoplasm and nucleus as reported in bronchial epithelial cells [41]. Nevertheless, our future study will further investigate the mechanism underlying cellular uptake of exogenous Sirt1. Other transcription factors such as zinc finger transcriptional factors Snail [42], Slug [43], and basic helix-loop-helix transcription factor Twist [44] are involved in the EMT process but C/EBPβ was used because of its reported role in the mesenchymal compartment in pulmonary fibrosis. Notwithstanding, our not-too distant future work will comprehensively evaluate the involvement of other transcription factors of EMT in IPF pathophysiology. We further observed that the deacetylation of C/EBPβ successfully limited α-SMA expression and collagen-I deposition. This inhibition of C/EBPβ binding to the α-SMA promoter correlates with the suppressive effect of SIRT1 in the A549 cells. Notably, this is the first study to suggest the EMT and fibrosis-promoting roles of C/EBPβ acetylation in A549 cells were attenuated by its deacetylation. Besides, we have examined the siRNA and TGFβ on normal epithelial cell (BEAS-2B, Cat. SCSP-5067, which possessed similar regulation of C/EBPβ binding to the α-SMA with A549 (Additional file 6: Fig. S6). However, more relevant research should be done in non-cancerous› alveolar epithelial cells to elimination effects of the tumor cells in the future.

## Conclusions

The present study showed that TGF-β could induce EMT, as evident from α-SMA expression in A549 cells, via C/EBPβ activation. Furthermore, acetylation of C/EBPβ could be a necessary step for binding to the α-SMA gene promoter and collagen-I deposition. Deacetylation of C/EBPβ could serve as an effective approach for restraining EMT and lung fibrosis. Future work will be conducted using in vivo experiments coupled with evaluation of more epithelial biomarkers to assess the impact of C/EBPβ modulation on EMT or pulmonary fibrosis.

## Supplementary Information

**Additional file 1: Fig S1.** TGF-β induced EMT and deposition of collagen-I in A549 cells. (a) A549 cells were exposed to TGF-β (0-20 ng/mL) for 24 h. (b) Expression of α-SMA and E-cadherin in A549 cells treated with TGF-β (10 ng/mL) for 0, 6, 12, 24, and 48 h was assessed using western blotting. Quantification of western blots were calculated according to gay analysis. (Referring Fig. 1a and b).

**Additional file 2: Fig S2.** Up-regulation of C/EBPβ is involved in TGF-β-induced EMT. (c) TGF-β increased C/EBPβ protein levels in A549 cells in a concentration- (0, 1, 2.5, 5, 10, and 20 ng/mL) dependent manner. (d) C/EBPβ expression increased gradually by 7.05 ± 0.81 folds by 24 h when TGF-β treatment was prolonged. (Referring Fig. 2a).

**Additional file 3: Fig S3.** TGF-β up-regulated the expression of α-SMA via C/EBPβ activation in A549 cells. TGF-β increased α-SMA protein levels via C/EBPβ activation. A549 cells were treated with TGF-β (10 ng/mL) for 24 h with or without the presence of C/EBPβ siRNA (10 nM) for 8 h. (Referring Fig. 3a).

**Additional file 4: Fig S4.** Involvement of C/EBPβ acetylation in TGF-β-induced EMT in A549 cells.

**Additional file 5: Fig S5.** Roles of C/EBPβ deacetylation in TGF-β-induced EMT and collagen deposition. (a) A549 cells were treated with TGF-β (10 ng/mL) for 24 h. (Referring Fig. 6a).

**Additional file 6: Fig S6.** BEAS-2B cells were treated with TGF-β (10 ng/mL) for 24 h.

## Data Availability

The data sets supporting the results of this article are included within the article and its additional files.

## Abbreviations

C/EBPβ: CCAAT/enhancer binding protein β
α-SMA: Alpha-smooth muscle actin
EMT: Epithelial-to-mesenchymal transition
IPF: Idiopathic pulmonary fibrosis
TGF-β: Transforming growth factor-beta
SIRT1: Sirtuin 1
ChIP: Chromatin immunoprecipitation
EMSA: Electrophoresis mobility shift assay
Co-IP: Co-immunoprecipitation
UIP: Usual interstitial pneumonia
ECM: Extracellular matrix
AECs: Alveolar epithelial cells
MAPK: Mitogen-activated protein kinase
PI3K: Phosphatidylinositol 3-kinase
ECL: Enhanced chemiluminescence
RT-PCR: Real time-polymerase chain reaction
ELISA: Enzyme-linked immunosorbent assay
HDAC: Histone deacetylase
CTGF: Connective tissue growth factor
ALI: Acute lung injury
