# Correction to: TGF‑β‑induced α‑SMA expression is mediated by C/EBPβ acetylation in human alveolar epithelial cells

**DOI:** 10.1186/s10020-021-00340-0

**Published:** 2021-07-12

**Authors:** Hui Ding, Jinjun Chen, Jingping Qin, Ruhua Chen, Zili Yi

**Affiliations:** 1 College of Bioscience and Biotechnology, Hunan Agricultural University, Changsha, 410128 Hunan China; 2 Department of Pulmonary and Critical Care Medicine, Yixing People Hospital, Affiliated to Jiangsu University, Yixing, 214200 Jiangsu China

## Correction to: Mol Med (2021) 27:22 https://doi.org/10.1186/s10020-021-00283-6

Following publication of the original article (Ding et al. 2021), due to a typesetting error, author Ruhua Chen has been missed to be marked as corresponding author.

The author group has been updated above and the original article (Ding et al. 2021) has been corrected.
