# Supplementary figures and images for: TGF-β-induced α-SMA expression is mediated by C/EBPβ acetylation in human alveolar epithelial cells
Source: Mol Med. 2021 Mar 4;27:22. doi: 10.1186/s10020-021-00283-6 (PMC7934236; doi:10.1186/s10020-021-00283-6)

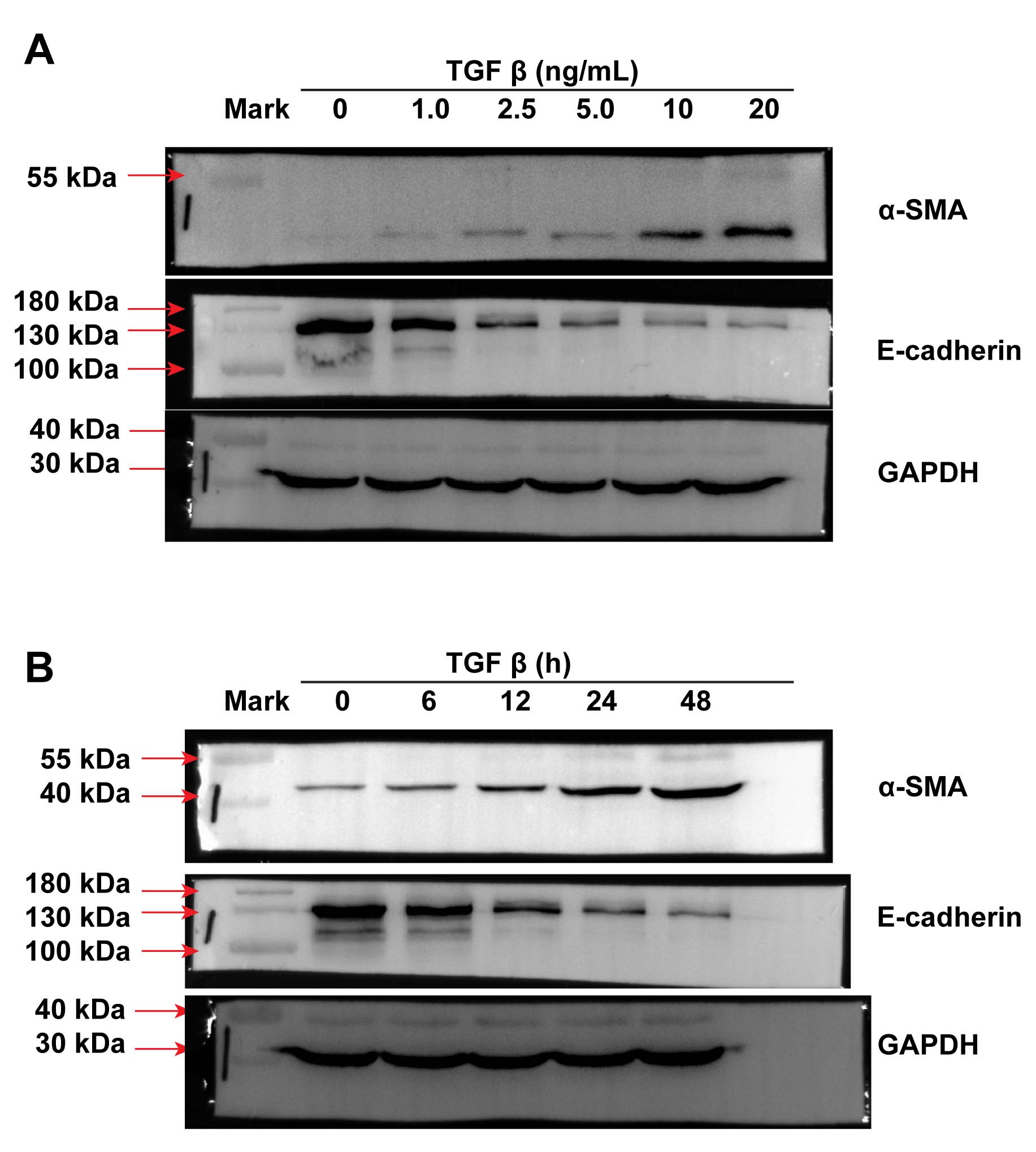

Supplement: Supplementary file 1 — Additional file 1: Fig S1. TGF-β induced EMT and deposition of collagen-I in A549 cells. (a) A549 cells were exposed to TGF-β (0-20 ng/mL) for 24 h. (b) Expression of α-SMA and E-cadherin in A549 cells treated with TGF-β (10 ng/mL) for 0, 6, 12, 24, and 48 h was assessed using western blotting. Quantification of western blots were calculated according to gay analysis. (Referring Fig. 1a and b). [file 10020_2021_283_MOESM1_ESM.jpeg]

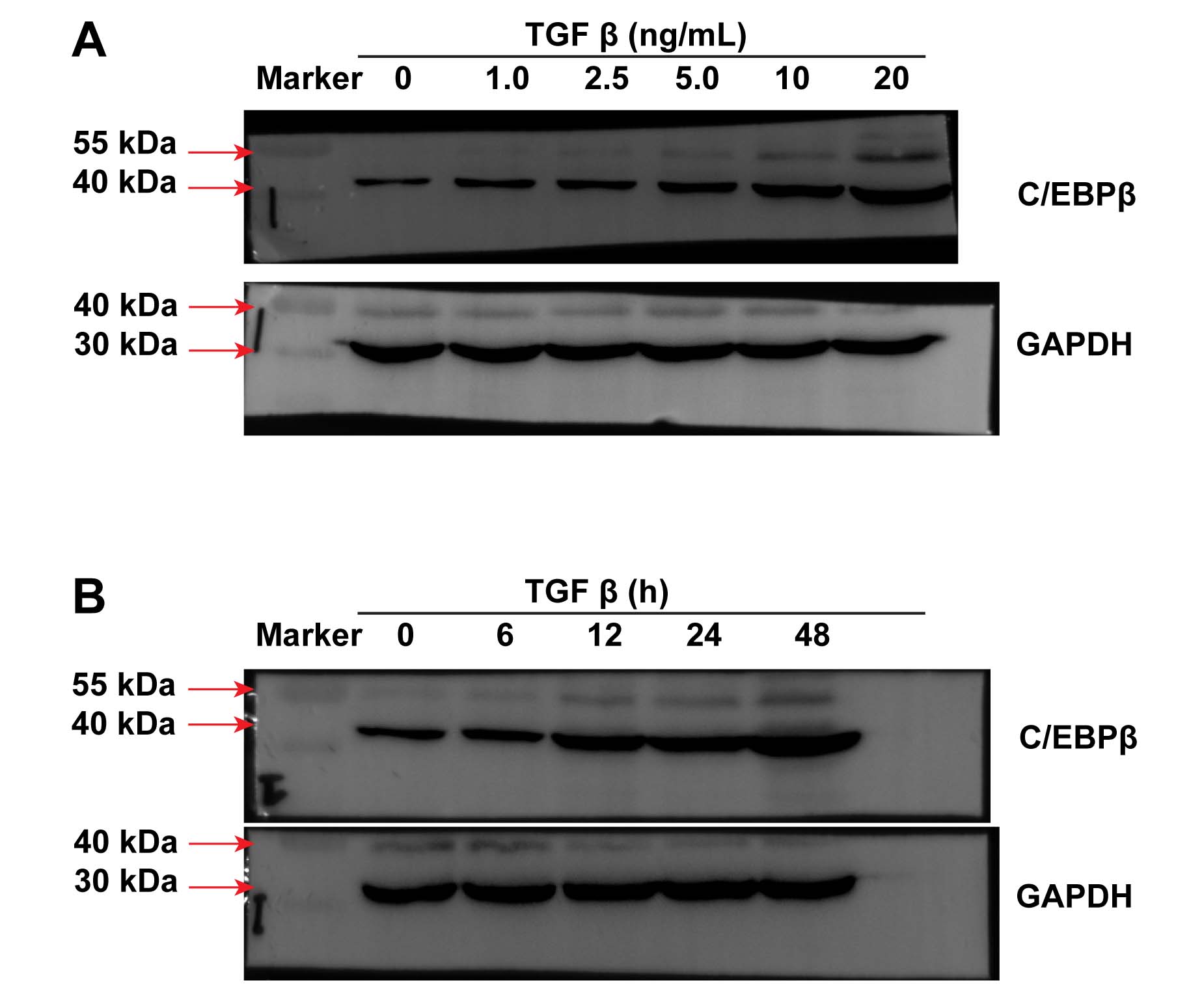

Supplement: Supplementary file 2 — Additional file 2: Fig S2. Up-regulation of C/EBPβ is involved in TGF-β-induced EMT. (c) TGF-β increased C/EBPβ protein levels in A549 cells in a concentration- (0, 1, 2.5, 5, 10, and 20 ng/mL) dependent manner. (d) C/EBPβ expression increased gradually by 7.05 ± 0.81 folds by 24 h when TGF-β treatment was prolonged. (Referring Fig. 2a). [file 10020_2021_283_MOESM2_ESM.jpeg]

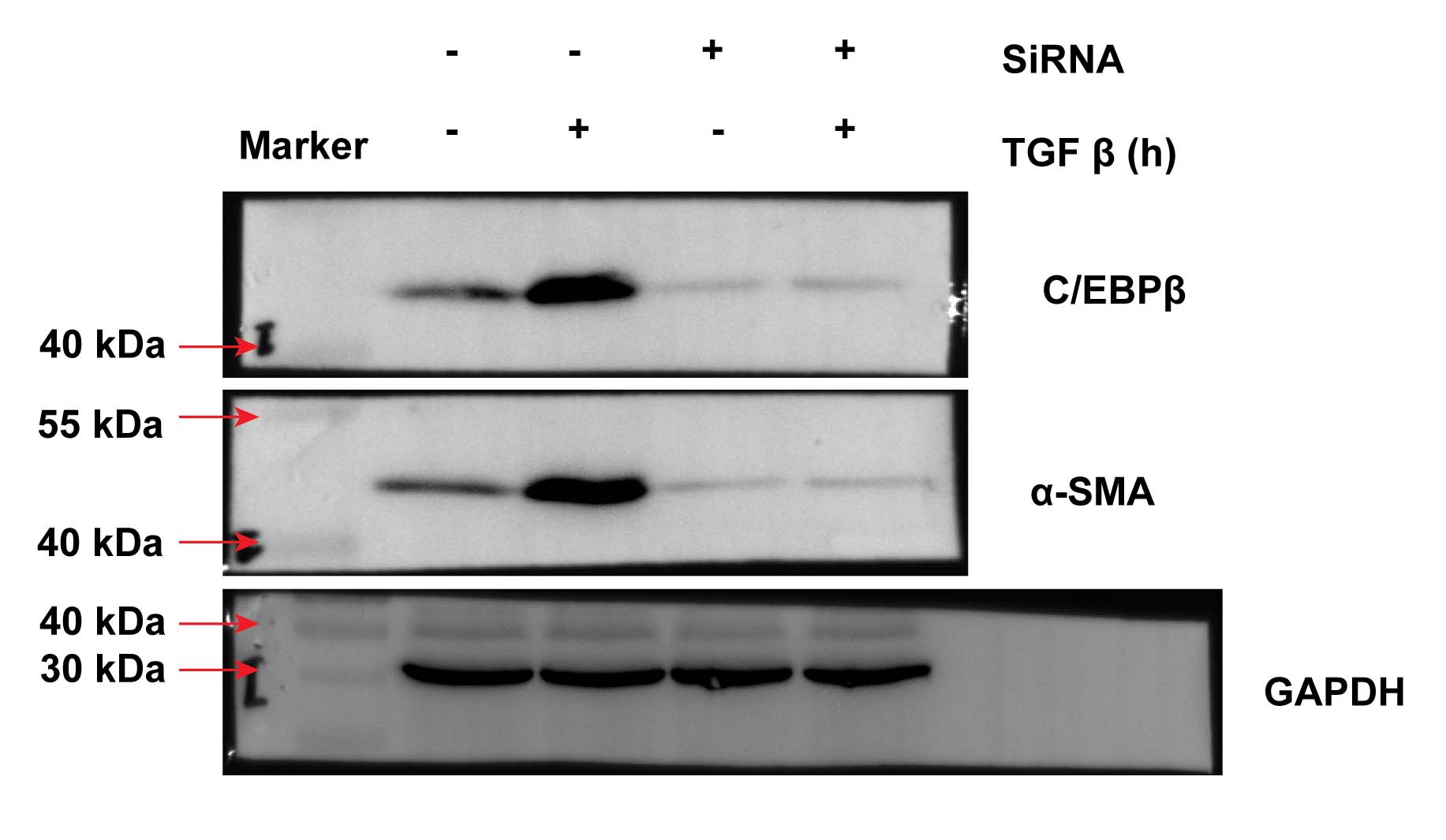

Supplement: Supplementary file 3 — Additional file 3: Fig S3. TGF-β up-regulated the expression of α-SMA via C/EBPβ activation in A549 cells. TGF-β increased α-SMA protein levels via C/EBPβ activation. A549 cells were treated with TGF-β (10 ng/mL) for 24 h with or without the presence of C/EBPβ siRNA (10 nM) for 8 h. (Referring Fig. 3a). [file 10020_2021_283_MOESM3_ESM.jpeg]

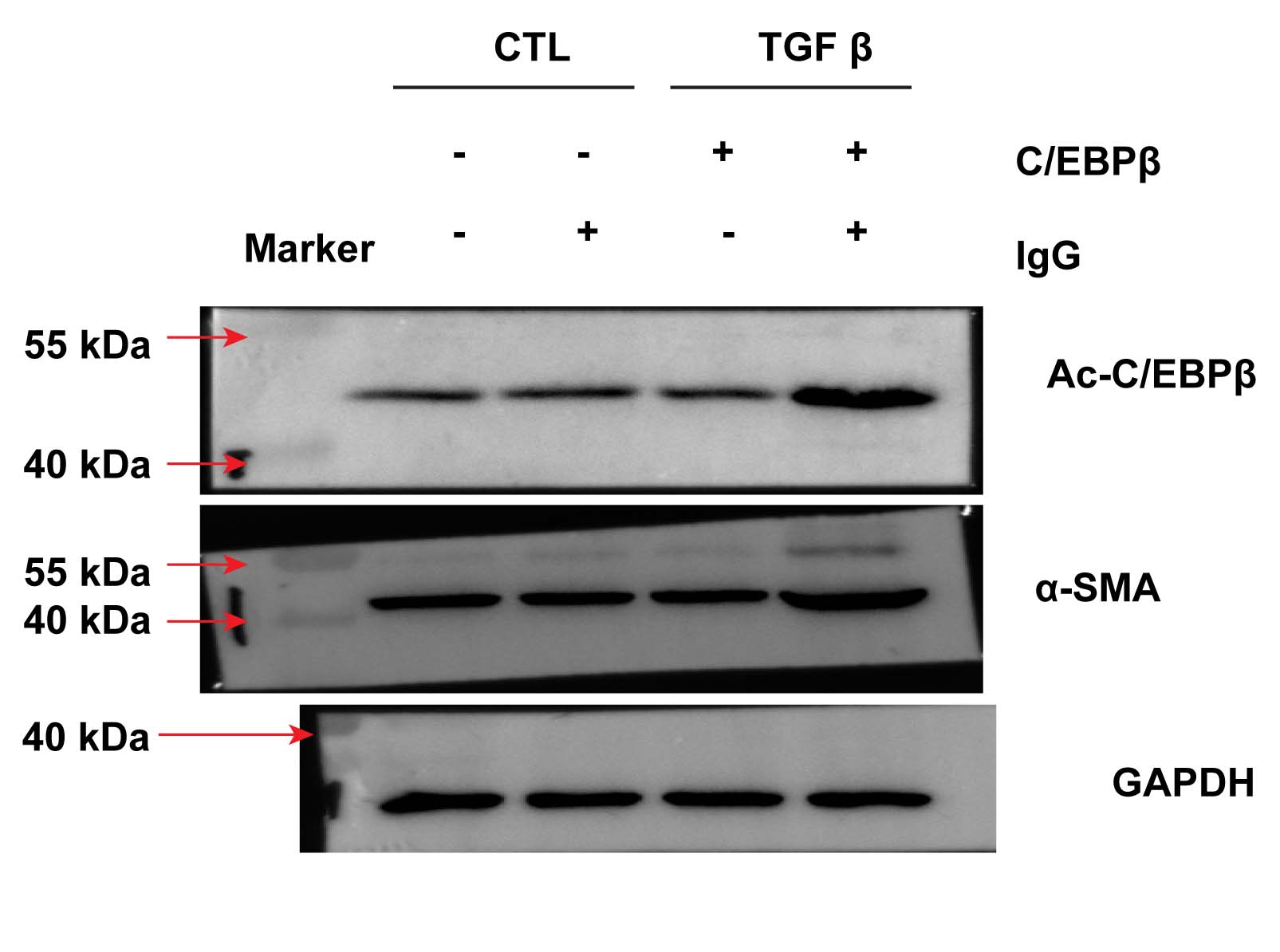

Supplement: Supplementary file 4 — Additional file 4: Fig S4. Involvement of C/EBPβ acetylation in TGF-β-induced EMT in A549 cells. [file 10020_2021_283_MOESM4_ESM.jpeg]

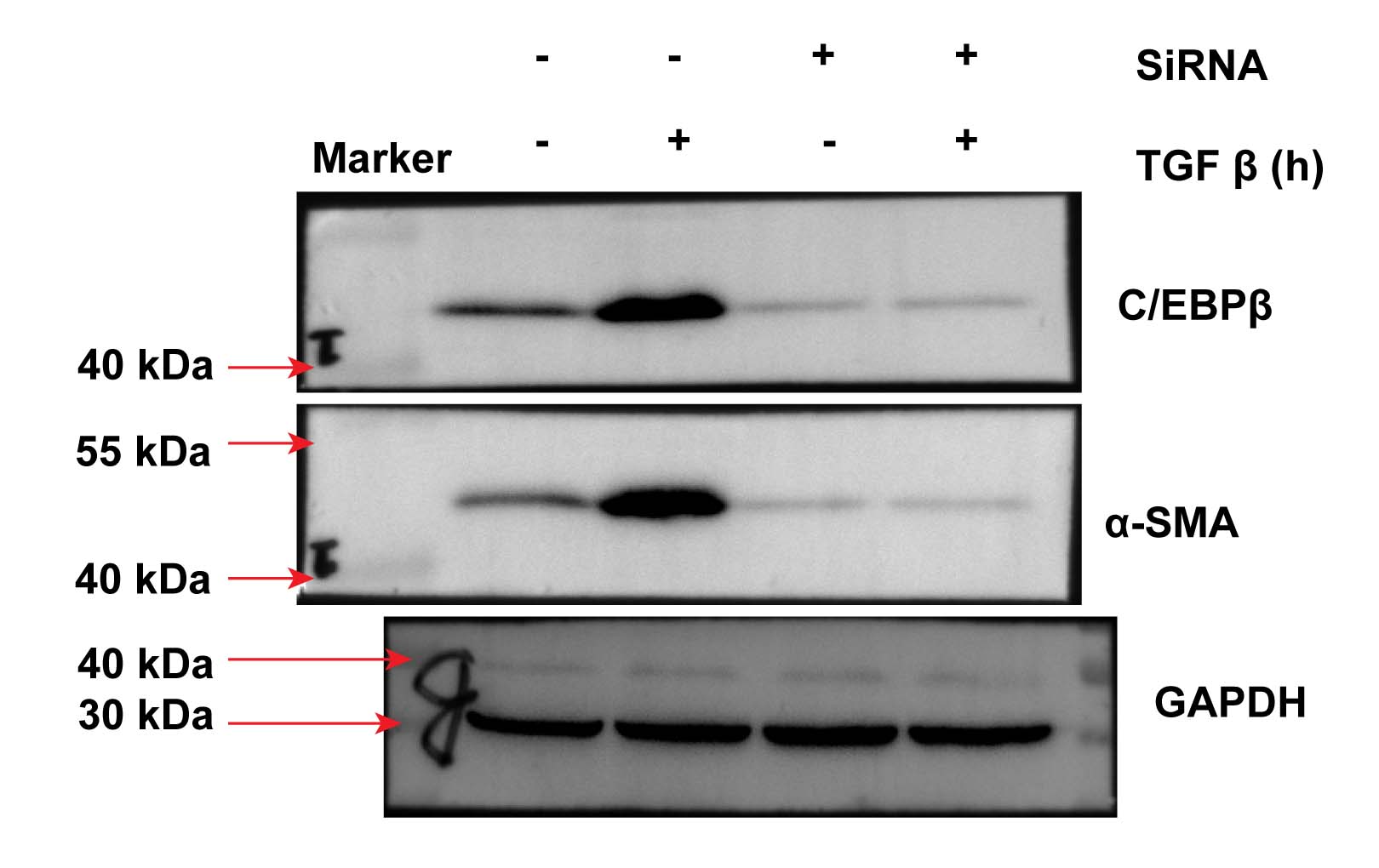

Supplement: Supplementary file 5 — Additional file 5: Fig S5. Roles of C/EBPβ deacetylation in TGF-β-induced EMT and collagen deposition. (a) A549 cells were treated with TGF-β (10 ng/mL) for 24 h. (Referring Fig. 6a). [file 10020_2021_283_MOESM5_ESM.jpeg]

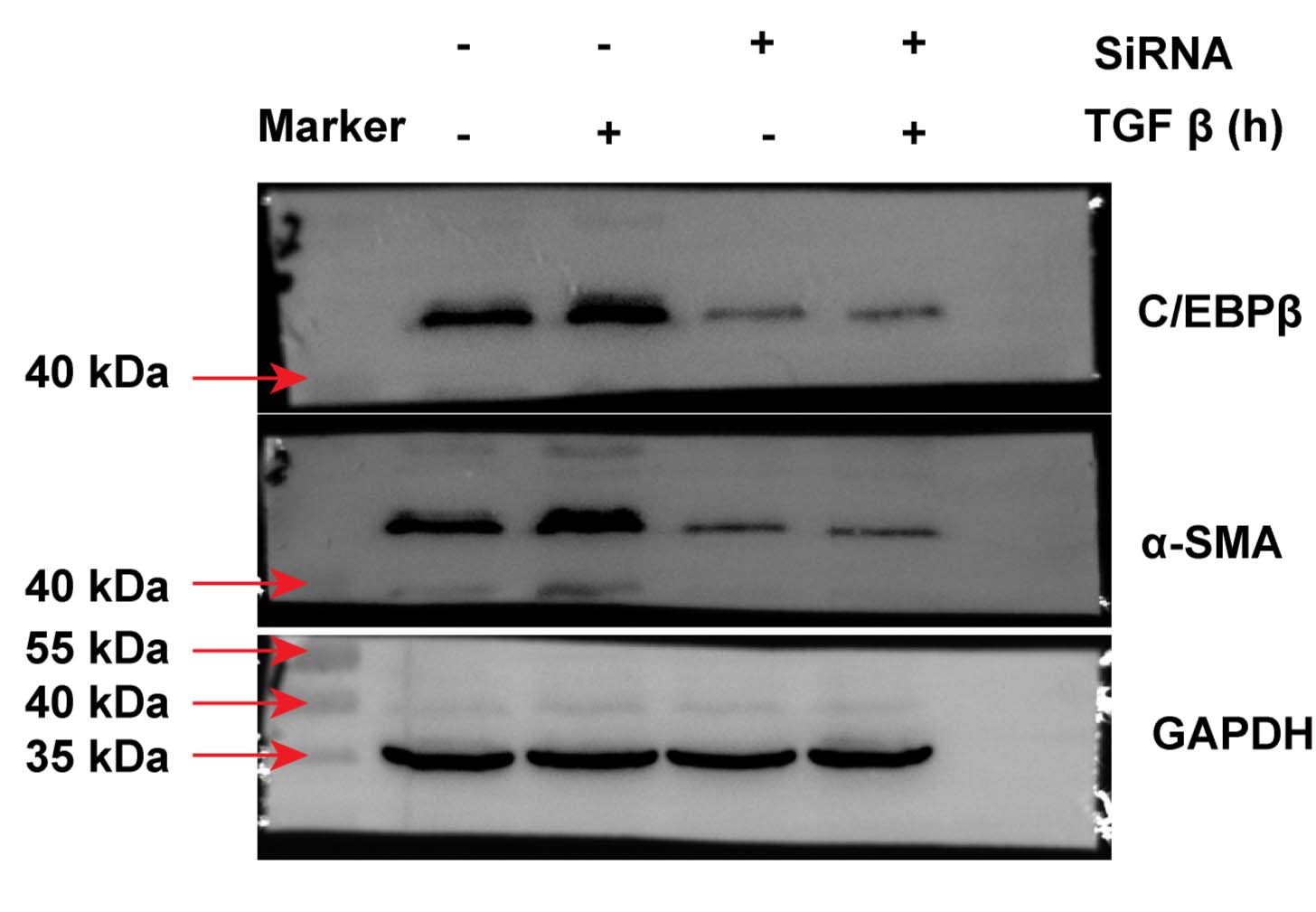

Supplement: Supplementary file 6 — Additional file 6: Fig S6. BEAS-2B cells were treated with TGF-β (10 ng/mL) for 24 h. [file 10020_2021_283_MOESM6_ESM.jpeg]
